# 

**Published:** 1907-08

**Authors:** 


### Contributors to Vol. LXIII.

### AUGUST, 1907—JULY, 1908.

---

Bainbridge, William S....New York
Bock, Franklin W....Rochester
Bryant, Joseph D....New York
Boswell, Charles O....Rochester
Bovee, J. Wesley....Washington
Cannaday, John E....Hansford, W. Va.
Cheesman, William S....Auburn
Colegrove, B. H....Buffalo
Cott, George F....Buffalo
Crothers, T. D....Hartford
Curtis, John G....New York
Daly, James R. L....New York
Dowd, J. Henry....Buffalo
Fairchild Bros. & Foster....New York
Frye, Maud J....Buffalo
Gardner, James A....Buffalo
Gould, George M....Philadelphia
Gram, Franklin C....Buffalo
Greenleaf, C. A....Canoga
Hayd, Herman E....Buffalo
Jenks, Jeremiah W....Ithaca
Jewett, Charles Sherman....Buffalo
Jones, Allen A....Buffalo
Jones, William B....Rochester
Judson, Adoniram B....New York
Krauss, William C....Buffalo
LeBreton, Prescott....Buffalo
Lewis, F. Park....Buffalo
Little, F. H....Muscatine, Ia.
MacDonald, Arthur....Washington
MacLeod, James A....Buffalo
MacLeod, Norman K....Buffalo
Manton, Walter P....Detroit
McKee, E. S....Cincinnati
McMurtry, Lewis S....Louisville

McPhedran, Alexander . . . . . . . . . . Toronto
Meisenbach, Roland O . . . . . . . . . . Buffalo
Monell, S. H . . . . . . . . . . New York
O'Reilly, R. M . . . . . . . . . . Washington
Parmenter, Frederick J . . . . . . . . . . Buffalo
Fitch, William E . . . . . . . . . . New York
Porter, Eugene H . . . . . . . . . . Albany
Ricketts, Benjamin M . . . . . . . . . . Cincinnati
Schering & Glatz . . . . . . . . . . New York
Shurly, E. L . . . . . . . . . . Detroit
Smith, Lee H . . . . . . . . . . Buffalo
Stockton, Charles G . . . . . . . . . . Buffalo
Van Peyma, P. W . . . . . . . . . . Buffalo
Walcott, Charles D . . . . . . . . . . Washington
Wende, Ernest . . . . . . . . . . Buffalo
Wende, Grover W . . . . . . . . . . Buffalo
Wilson, Nelson W . . . . . . . . . . Buffalo
Williams, Herbert U . . . . . . . . . . Buffalo
Wyman, Walter . . . . . . . . . . Washington

Medical Association of Central New York
Medical Society County of Erie
Roswell Park Medical Club

### BOOKS RECEIVED.

Diseases of the Stomach. By Dr. I. Boas, Berlin, Germany. Authorised English-American Edition by Albert Bernheim, M.D. (Freiburg, Germany), Assistant to the late Dr. D. D. Stewart at the Philadelphia Polyclinic Hospital and Post-graduate School, as Instructor in the Department of Diseases of the Stomach asd Intestines. Illustrated with five full-page plates and sixty-five engravings in the text. 730 royal octavo pages. Philadelphia: F. A. Davis Company. (Cloth $5.50; half morocco, $7.00).

---

Diseases of Infancy and Childhood. Their Dietetic, Hygienic, and Medical Treatment. A Textbook designed for Practitioners and

Students in Medicine. By Louis Fischer, M.D., Visiting Physician to The Willard Parker and Riverside Hospitals, New York. With 303 text illustrations, several in colors, and twenty-seven full-page half-tone and color plates. 979 royal octavo pages. Philadelphia: F. A. Davis Company. (Cloth $6.50; half morocco $8.00, net prices.)

---

The Practice of Obstetrics. By American Authors. Edited by Charles Jewett, M.D., Professor of Obstetrics in the Long Island College Hospital, Brooklyn, N. Y. In one handsome octavo volume of 786 pages, with 445 engravings in black and colors and 36 full-page colored plates. New York and Philadelphia: Lea Brothers & Co. (Cloth, $5.00; leather, $6.00; half morocco, $6.50 net prices.)

---

International Clinics. A Quarterly of Illustrated Clinical Lectures and especially prepared articles on Treatment, Medicine, Surgery, Neurology, Pediatrics, Obstetrics, Gynecology, Orthopedics, Pathology, Dermatology, Ophthalmology, Otology, Rhinology, Laryngology, Hygiene and other topics of interest to students and practitioners. Edited by W. T. Longcope, M.D. Volume II, seventeenth series. Philadelphia and London: 1907. J. B. Lippincott Co. (Cloth $2.00).

---

A Textbook of Practical Therapeutics, with Especial Reference to the Application of Remedial Measures to Disease and their Employmest upon a Rational Basis. By Hobart Amory Hare, M.D., B.Sc., Professor of Therapeutics and Materia Medica in the Jefferson Medical College of Philadelphia. New (12th) edition, enlarged and thoroughly revised to accord with the eighth decennial revision of the U. S. Pharmacopeia. In one octavo volume of 939 pages, with 114 engravings and four colored plates. Philadelphia and New York: 1907. Lea Brothers & Co. (Cloth, $4.00; leather, $5.00; half morocco, $5.50, net prices.)

---

Modern Medicine. Its Theory and Practice. In Original Contributions by American and Foreign Authors. Edited by William Osler, M.D., Regius Professor of Medicine in Oxford University, England. Assisted by Thomas McCrea, M.D., Associate Professor of Medicine and Clinical Therapeutics in Johns Hopkins University, Baltimore. In seven octavo volumes of about 1,000 pages each; illustrated. Volume II. Philadelphia and New York: 1907. Lea Brothers & Co. (Cloth, $6.00; leather, $7.00; half morocco, $7.50, net prices.)

---

Practical Fever Nursing. By Edward C. Register, M.D., Professor of the Practice of Medicine in the North Carolina Medical College. Octavo, pp. 352. Illustrated. Philadelphia and London: 1907. W. B. Saunders Co. (Price, $2.50.)

---

Treatment of the Diseases of Children. By Charles Gilmore Kerley, M.D., Professor of Diseases of Children in the New York Polyclinic Medical School and Hospital. Octavo, pp. 597. Illustrated. Philadelphia and London: 1907. W. B. Saunders Co. (Price, $5.00.)

---

A Pocket Textbook of Materia Medica, Therapeutics, Prescription Writing, Medical Latin and Medical Pharmacy. By William Schleif, Ph.G., M.D., University of Pennsylvania, Philadelphia. Third edition, 12mo., 470 pages. Philadelphia asd New York: 1907. Lea Brothers & Co. (Cloth, $2.50, net.)

American Edition of Nothnagel's Practice. Diseases of the Intestines and Peritoneum. Second edition. Authorised translation under the editorial supervision of Alfred Stengel, M.D., Philadelphia. Philadelphia and London: 1907. W. B. Saunders Co. (Price, cloth, $5.00; sheep or half morocco, $6.50.)

---

Diseases of the Rectum. Their Consequences and Nonsurgical Treatment. By W. C. Brinkerhoff, M.D. 12 mo., pp. 207. Chicago: Orban Publishing Co. (Price, $2.00.)

---

Report of the Commissioner of Education for the year ending June 30, 1905. Vo. 2. Washington: Government Printing office.

---

Twenty-sixth Annual Report of the State Department of New York for the year ending December 31, 1905. Albany: Brandow Printing Co.

